# Role of *X11* and *ubiquilin* as In Vivo Regulators of the Amyloid Precursor Protein in *Drosophila*

**DOI:** 10.1371/journal.pone.0002495

**Published:** 2008-06-25

**Authors:** Garrett G. Gross, R. M. Renny Feldman, Atish Ganguly, Jinhui Wang, Hong Yu, Ming Guo

**Affiliations:** 1 Department of Neurology, The David Geffen School of Medicine, University of California Los Angeles, Los Angeles, California, United States of America; 2 Department of Pharmacology, The David Geffen School of Medicine, University of California Los Angeles, Los Angeles, California, United States of America; 3 Department of Brain Research Institute, The David Geffen School of Medicine, University of California Los Angeles, Los Angeles, California, United States of America; University of Florida, United States of America

## Abstract

The Amyloid Precursor Protein (APP) undergoes sequential proteolytic cleavages through the action of β- and γ-secretase, which result in the generation of toxic β-amyloid (Aβ) peptides and a C-terminal fragment consisting of the intracellular domain of APP (AICD). Mutations leading to increased APP levels or alterations in APP cleavage cause familial Alzheimer's disease (AD). Thus, identification of factors that regulate APP steady state levels and/or APP cleavage by γ-secretase is likely to provide insight into AD pathogenesis. Here, using transgenic flies that act as reporters for endogenous γ-secretase activity and/or APP levels (GAMAREP), and for the APP intracellular domain (AICDREP), we identified mutations in *X11L* and *ubiquilin (ubqn)* as genetic modifiers of APP. Human homologs of both *X11L* (*X11/Mint*) and *Ubqn* (*UBQLN1*) have been implicated in AD pathogenesis. In contrast to previous reports, we show that overexpression of *X11L* or human *X11* does not alter γ-secretase cleavage of APP or Notch, another γ-secretase substrate. Instead, expression of either *X11L* or human *X11* regulates APP at the level of the AICD, and this activity requires the phosphotyrosine binding (PTB) domain of X11. In contrast, Ubqn regulates the levels of APP: loss of *ubqn* function leads to a decrease in the steady state levels of APP, while increased *ubqn* expression results in an increase in APP levels. Ubqn physically binds to APP, an interaction that depends on its ubiquitin-associated (UBA) domain, suggesting that direct physical interactions may underlie Ubqn-dependent regulation of APP. Together, our studies identify X11L and Ubqn as in vivo regulators of APP. Since increased expression of X11 attenuates Aβ production and/or secretion in APP transgenic mice, but does not act on γ-secretase directly, X11 may represent an attractive therapeutic target for AD.

## Introduction

**Figure 1:**
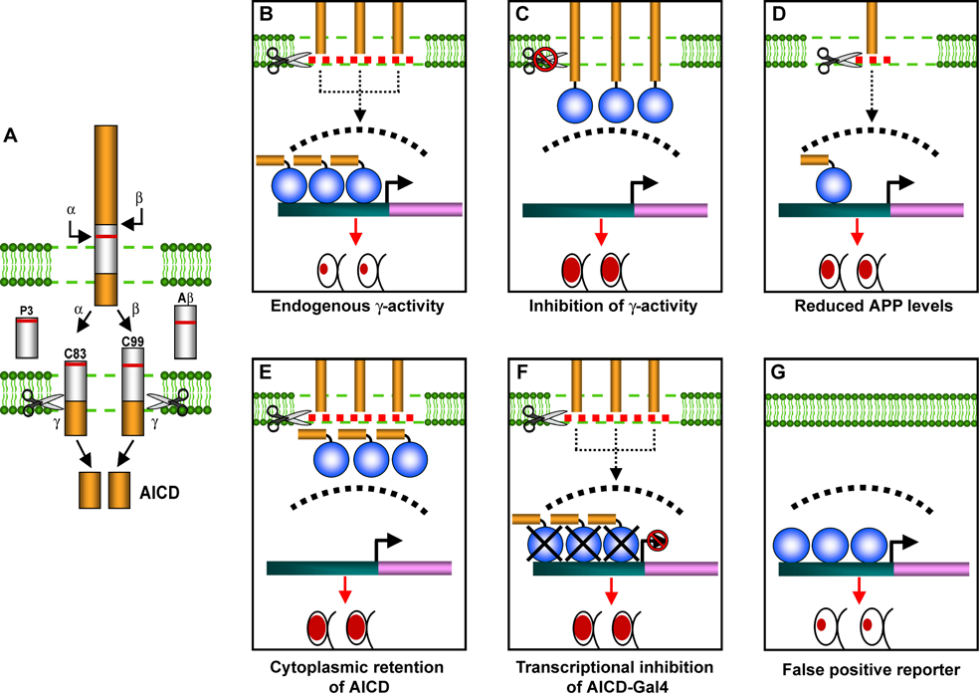
Schematics depicting sites of APP cleavage and GAMAREP. (A) The sequential actions of either α-secretase or β-secretase, which cleaves APP in its extracellular domain, and γ-secretase, which cleaves APP within its transmembrane domain, generate AICD and C83 or C99, respectively. α-secretase cleaves APP within the sequence of Aβ, thus precluding the generation of Aβ. (B) GAMAREP (GMR-C99-Gal4, UAS-*grim*) contains two components: First, C99-Gal4, a chimeric protein with an N-terminal cleavable signal sequence, transmembrane and intracellular domain (yellow bar) and a C-terminal Gal4 (blue circle) is specifically expressed in the eye and is a substrate for γ-secretase. Second, Grim (purple bar) is expressed under the control of a Gal4-dependent promoter (green bar) as a readout. In the presence of endogenous γ-secretase activity (scissor), the unleashed AICD-Gal4 (yellow bar and blue circle fragment) translocates to the nucleus (dashed curve) and binds to the UAS element and activates (black arrow pointing to the right) Grim expression. This leads to apoptosis in the eye, resulting in flies with small and rough eyes (red ovals depict relative eye size). (C) In the absence of γ-cleavage, Gal4 remains tethered at the membrane and therefore is unable to activate Grim expression. This results in flies with increased eye size (i.e., suppression of GAMAREP phenotype). (D) When APP (C99) levels are downregulated, fewer substrate molecules are available (illustrated as one C99 molecule rather than three in B) to activate Grim-dependent apoptosis. This results in larger (suppressed) eye size. (E) When AICD is retained in the cytoplasm, or its levels or function are reduced (not illustrated here), apoptosis occurs less efficiently and the eyes remain large. (F) When AICD-Gal4 mediated transcription is inhibited, apoptosis is also reduced, resulting in flies with larger (suppressed) eyes. In summary, GAMAREP allows the identification of factors that regulate endogenous γ-secretase activity (as in C), as well as factors that regulate APP steady state levels (as in D), and AICD function, stability and/or cytoplasmic retention (as in E and F). (G) The false positive reporter. Suppression of GAMAREP can also arise from factors that regulate processes other than APP biology, such as mutations modifying GMR- or Gal4-dependent transcriptional activation, or apoptosis, which we collectively named false positives. To eliminate these factors, we employed a “false positive” reporter (GMR-Gal4, UAS-apoptotic gene). True modifiers are those that modify they eye phenotypes of GAMAREP, but not those of the false positive reporter.

One of the pathological hallmarks of Alzheimer's disease (AD) is the accumulation of amyloid plaques consisting of toxic β-amyloid (Aβ) peptides. These peptides arise from the sequential cleavage of the Amyloid Precursor Protein (APP), a type I transmembrane protein, by two proteases known as β- and γ-secretase (Fig. 1A). APP proteolysis by β-secretase generates an APP C-terminal fragment (CTF) known as C99. Subsequent cleavage of C99 by γ-secretase results in the release of Aβ into the lumen and the APP intracellular domain (AICD) into the cytosol, where it can contribute to a transcriptional regulatory complex [1]. In addition to this amyloidogenic pathway, APP can also undergo non-amyloidogenic processing via sequential cleavage by α- and γ- secretase (Fig. 1A). α-secretase cleaves within the Aβ sequence, thereby precluding the formation of Aβ. α-cleavage produces an APP CTF known as C83, which also serves as a substrate for γ-secretase activity [1].

γ-secretase activity resides in a multi-protein complex that minimally contains Presenilins, Nicastrin, Aph-1 and Pen-2 [2], [3]. Mutations in APP, Presenilin 1 and Presenilin 2 cause familial, early onset AD [4]–[7]. In addition, the triplication of the APP locus as well as promoter mutations in APP that increase APP expression are associated with AD [8], [9]. The function of the AICD may also be crucial for AD pathogenesis, since each time Aβ is generated, AICD is simultaneously released. AICD, in conjunction with two PTB domain-containing proteins (Fe65 and Tip60), can enter the nucleus and regulate the transcription of target genes, including APP itself [10]. In addition, AICD has also been implicated in other processes including cell signaling, apoptosis and calcium homeostasis [11]–[21]. Therefore, identifying genes that regulate APP steady-state levels, APP cleavage, and the fate and activity of AICD are likely to further our understanding of AD pathogenesis.

The X11/Mint protein family consists of three mammalian members: X11α and X11β, which are expressed in neurons, and X11γ which is ubiquitously expressed. All three X11 proteins contain a phosphotyrosine binding (PTB) domain, followed by two PSD95/Dlg/ZO-1 (PDZ) domains [22]. Several observations suggest links between X11 and AD. First, X11α and X11β have been found in amyloid plaques in post-mortem AD brains [23], [24]. Second, increased X11α and X11β expression in mammalian cells leads to a reduced secretion of extracellular Aβ [25]–[27], while transgenic mice expressing either X11α or X11β are associated with reduced levels of Aβ [28], [29]. Third, X11 proteins physically interact with AICD via their PTB domains [30], [31] and inhibit AICD-dependent transcription [32]. Fourth, X11α and X11β overexpression increases APP steady-state levels both *in vitro* and *in vivo*, likely due to altered maturation in the secretory pathway or endocytic trafficking of APP [25], [27], [33], [34]. Finally, the X11 proteins bind to presenilin via their PDZ domains [35]. Furthermore, X11α and X11β have been reported to modulate γ-secretase in mammalian cells [36]; however, alterations in the levels of C83 and C99 are not observed in transgenic mice overexpressing X11 [29]. These conflicting observations leave it unclear whether X11 overexpression can regulate γ−secretase activity.

*Ubiquilin 1 (UBQLN1)* is another gene that has been linked to AD. *UBQLN1* encodes a protein with ubiquitin-like (UBL) and ubiquitin-associated (UBA) domains, as well as Sti1 repeats [37], which are often associated with chaperone activity [38]. Several studies suggest links between *UBQLN1* and AD. First, in post-mortem AD brains, UBQLN1 is found in neurofibrillary tangles [37], a pathological hallmark of AD along with amyloid plaques [1]. Second, the genomic region containing *UBQLN1*, 9q22, has been identified as containing one major candidate gene for conferring a predisposition to late onset AD [39], [40]. Some reports [41], [42], but not others [43]–[46], suggest that genetic variants in the *UBQLN1* gene, including one known as *UBQ-8i* that deletes one Sti1 repeat, are associated with increased risk for the more prevalent late-onset forms of AD. Further evidence that UBQ-8i has enhanced toxicity comes from studies in *Drosophila* demonstrating that expression of human *UBQ-8i* in flies leads to earlier onset and more severe eye degeneration than does expression of wildtype human *UBQLN1* [47]. Third, UBQLN1 binds PS1 and PS2 [37], and the fly homolog of Presenilin binds to both human and fly Ubiquilin (Ubqn), respectively [47]. *Drosophila ubqn* antagonizes *presenilin* (*psn*) function in both loss-of-function and gain-of-function studies during development and adult-onset neurodegeneration in vivo [47], [48]. Finally, in cultured cells, downregulation of *UBQLN1* expression alters APP levels and Aβ secretion by modulating APP trafficking [49]. However, reports of altered *UBQLN1* expression on APP processing are conflicting [48]–[50]. Because increases in APP levels and/or altered APP processing are likely to be important risk factors for AD, it is crucial to clarify the effect of UBQLN1 on APP in an in vivo system. Additionally, since both X11 and UBQLN1 have been implicated as regulators of APP steady state levels and APP processing, it is crucial to assess the extent to which each molecule contributes to the myriad regulations of APP-related processes, and to determine what their net effect is in vivo.

*Drosophila melanogaster* is a useful model organism for studying neurodegenerative diseases and AD-related processes [51]–[54]. *Drosophila* contains homologs of APP [55] and all four components of the γ-secretase complex [56], [57]. Expression of human APP in *Drosophila* results in its cleavage by endogenous α- and γ-secretase activities [58], [59]. Furthermore, co-expression of human β-secretase (BACE1) and human APP fully reconstitutes APP β-cleavage [60]. In addition, *Drosophila* contains homologs of X11/Mint family proteins [22], [61] and UBQLN1 with evolutionarily conserved domain architecture [47], [48].

We engineered transgenic flies that act as living reporters for γ-secretase activity in the eye [59] (Fig 1B-F). These flies (GMR-C99-Gal4, UAS-*grim*), known as gamma-secretase activity and APP level reporters (hereafter called GAMAREP), express a chimeric protein which contains a signal sequence, followed by a fusion of the C99 fragment of APP to the yeast transcription factor Gal4. This C99-Gal4 protein is expressed specifically in the developing eye in the presence of a Gal4 responsive element that drives an apoptosis-inducing gene known as *grim* [62], [63]. In the presence of endogenous γ-secretase activity (Fig. 1B), AICD-Gal4 is liberated from the membrane, migrates to the nucleus and activates transcription of *grim*. The resulting apoptosis generates flies with small and rough eyes [59]. When γ-secretase activity is impaired (Fig. 1C), Gal4 remains tethered to the membrane, resulting in less apoptosis. Therefore, the size and roughness of the eye inversely correlates, in a semi-quantitative manner, with the level of endogenous γ-secretase activity (high levels of activity results in flies with small eyes while low levels are associated with nearly normal eyes). These flies act as sensitive reporters for modest changes (two-fold) in the levels of known γ-secretase components [59]. Though originally intended to identify regulators of γ-secretase activity [59], the eye phenotypes of GAMAREP flies are also expected to be sensitive to genetic perturbations that alter the levels of APP (Fig. 1D), AICD function, and/or the transcriptional activity of AICD-Gal4 (Fig. 1E, F). To discriminate factors that regulate transgene expression levels, Gal4-dependent transcriptional efficiency or apoptosis, we also generated a false positive reporter (GMR-Gal4, UAS-apoptotic gene) (Fig. 1G). True modifiers will be identified as factors that modify only GAMAREP eye phenotypes, and not those of the false positive reporter. Here we report the identification of *X11L* and *ubqn* as regulators of APP in *Drosophila*.

## Results

### Increased expression of *X11L* suppresses GAMAREP eye phenotypes

**Figure 2:**
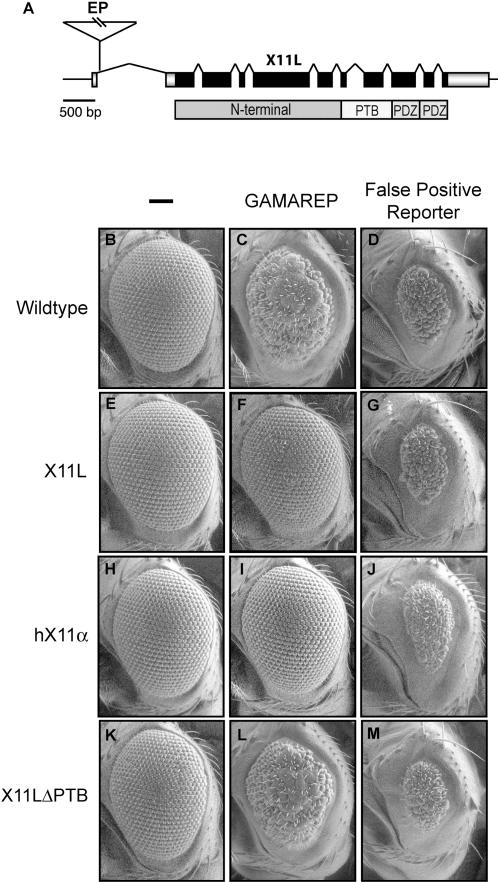
Overexpression of fly *X11L* or human *X11α* specifically suppresses GAMAREP. (A) Genomic map of *Drosophila X11L* (CG5675, cytological location 16B7). The P element insertion (triangle), *X11L* coding and the untranslated regions (dark and shaded rectangles, respectively) are illustrated. *X11L* encodes a protein with a PTB and two PDZ domains that are conserved in human X11 proteins. (B–M) Scanning electron micrographs (SEM) of adult fly eyes of various genotypes. GAMAREP flies exhibit small, rough eyes (C) as compared to the wildtype (B). Overexpression of *X11L* (GMR-*X11L*) shows no external eye phenotypes (E), but strongly suppresses GAMAREP eye phenotypes (F). Expression of human *X11α* (GMR-*X11α*), which by itself does not produce any visible eye phenotypes (H), suppresses GAMAREP phenotypes as effectively as fly *X11L* overexpression (I). *X11L*'s ability to suppress GAMAREP is dependent on its PTB domain, since overexpression of *X11L*ΔPTB (GMR-*X11L*ΔPTB) (K) fails to suppress GAMAREP eye phenotype (L). The ability of X11 to suppress GAMAREP eye phenotype is specific, since overexpression of X11L, X11ΔPTB or human X11 fails to suppress the eye phenotype of the false-positive reporter (GMR-Gal4, UAS-*grim/reaper*) (D, G, J and M). The *X11L*ΔPTB transgene expresses a truncated protein as detected by anti-X11L antibodies [75]. Of note, strong overexpression of *X11L* (multiple copies of GMR-*X11L* or GMR-Gal4, UAS-*X11L*) can result in a rough eye phenotype (data not shown), consistent with previous reports [76].

We carried out a genetic screen (to be described in detail elsewhere) in which GAMAREP flies were crossed with flies carrying single insertions of the EP P element on the X chromosome. EP elements carry a Gal4 responsive promoter pointing outwards from the end of the transposon. Thus, when inserted near the 5′ end of a gene, EP elements can drive nearby gene overexpression in a Gal4 dependent manner. From this screen, we identified one suppressor line, which contains an EP element insertion in the 5′ region of the *X11L* gene (CG5675) (Fig. 2A). To confirm that the GAMAREP suppression is due to overexpression of *X11L*, we showed that eye-specific overexpression of *X11L* (GMR-*X11L*) (Fig. 2E) indeed suppressed the GAMAREP small eye phenotype (Fig. 2F compared to C). *X11L* overexpression was confirmed using anti-X11L antibodies (Supplementary Fig. S1). Importantly, *X11L* overexpression had no effect on the small eye phenotypes of flies expressing apoptosis-inducing genes under the direct control of GMR-Gal4 (false positive reporters; [59]) (Fig. 2G compared to D), indicating that X11L acts specifically on C99, not on GAL4 or apoptotic genes.

To demonstrate that the mechanism we uncovered for *X11L* is shared by human *X11α,* we expressed human *X11α* specifically in the fly eye (Fig. 2H). Strikingly, expression of human *X11α* also suppressed the small eye phenotype of GAMAREP (Fig. 2I), but not that of the false positive reporter (Fig. 2J). This suggests that *Drosophila X11L* and human *X11α* show functional conservation and that mechanisms of action discovered in flies are likely to be relevant to humans.

### X11L overexpression regulates APP at the level of AICD

**Figure 3:**
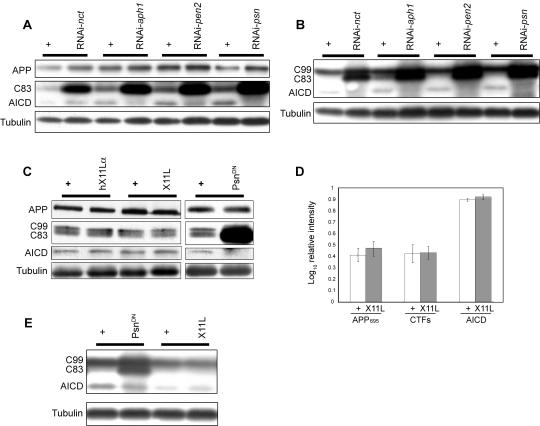
*X11L* overexpression does not alter APP levels or processing. (A–C, E) Western blotting of adult head lysates from various transgenic flies. (A) When APP-9Myc is expressed, the endogenous α- and γ- secretase mediated cleavages generate C83 and AICD, respectively. RNAi knockdown of any one of the four components of the γ-secretase complex results in reduction of AICD levels and an increase in the levels of C83, as compared with sibling controls. (B) When C99-9Myc is expressed, the endogenous γ-cleavage generates AICD. RNAi knockdown of any one of the four γ-secretase components results in a reduction in AICD levels and a simultaneous increase in levels of the CTFs. (C) Co-expression of human BACE and APP-9Myc generates C83, C99 and AICD. Overexpression of Psn^DN^ results in a reduction of AICD and an increase in the levels of γ-secretase substrates (C83 and C99). In contrast, expression of neither *X11L* nor human *X11α* results in any alteration in the CTF levels. (D) Western blot quantifications of APP and its fragments suggest that there are no significant differences in the levels of either full length APP or its fragments as compared to control. Values are normalized to the corresponding tubulin loading controls. Data from X11 overexpression (dark bars) and control (white bars) are analyzed by paired t-test. (E) When C99-9Myc is expressed, expression of Psn^DN^ results in a decrease in AICD levels and an increase in C83 levels, while overexpression of *X11L* fails to show any changes. Note that panels depicting CTFs are from the same Western blots exposed for different lengths of time than the corresponding APP panels. For each sample, sibling flies of the appropriate genotype raised under identical conditions were used as controls, and are shown immediately adjacent to the corresponding experimental sample (linked by a horizontal bar).

To determine how increased *X11L* expression regulates APP in the GAMAREP system, we established assays for APP levels and cleavage in *Drosophila*. Full length human APP (APP_695_) or C99 was myc-tagged at the C-terminus and expressed in the eye. Tissue lysates were examined by Western blotting with an anti-Myc antibody to follow the fate of APP (Fig. 3A) or C99 (Fig. 3B). APP-Myc or C99-Myc was cleaved by endogenous α- and γ-secretase in both systems, yielding an AICD-Myc fragment (γ-secretase cleaved) and C83 (an α-secretase cleaved fragment) (Fig. 3A and 3B). Furthermore, although no endogenous BACE activity has been definitively demonstrated in *Drosophila*, expression of human BACE in the presence of APP resulted in the generation of C99, in addition to C83 and AICD (Fig. 2C). RNAi-mediated silencing of any one of the four components of the γ-secretase complex, *psn*, *nct*, *aph-1* or *pen-2*, resulted in attenuated AICD generation and an increase in the levels of CTFs (γ-secretase substrates) (Fig. 3A and 3B). In addition, overexpression of a dominant negative form of psn (Psn^D279A^ which corresponds to human PS1^D257A^ [59]) resulted in similar effects on APP processing (Fig. 3C and E).

Since human X11α has previously been shown to bind Presenilin and regulate γ-secretase-mediated cleavage in mammalian cells [35], we asked if *Drosophila* X11L overexpression suppressed GAMAREP by inhibiting γ-secretase activity. We crossed flies overexpressing *X11L* with flies expressing APP-Myc, and their progeny were subjected to Western blot analysis. In contrast to what was observed for the knockdown of *psn*, *nicastrin (nct)*, *aph-1* or *pen-2* function, we did not see any reduction in AICD levels or any CTF accumulation (Fig. 3C and D). Similarly, flies overexpressing *X11L* in the background of C99-Myc expression also failed to show any evidence of γ-secretase inhibition (Fig. 3E). These results suggest that the predominant effect of *X11L* overexpression in vivo is not due to γ-secretase inhibition.

With increased expression of *X11L* (multiple copies of transgenes), we observed a slight increase in the steady-state levels of APP (data not shown), which is consistent with reported findings in mammals [25], [27], [33], [34]. However, full-length APP and its proteolytic fragments increased largely in proportion. The slight increase in levels of AICD is likely to be physiologically inconsequential, since elevated levels of AICD are predicted to enhance the GAMAREP eye phenotype, whereas we observed a robust suppression.

**Figure 4:**
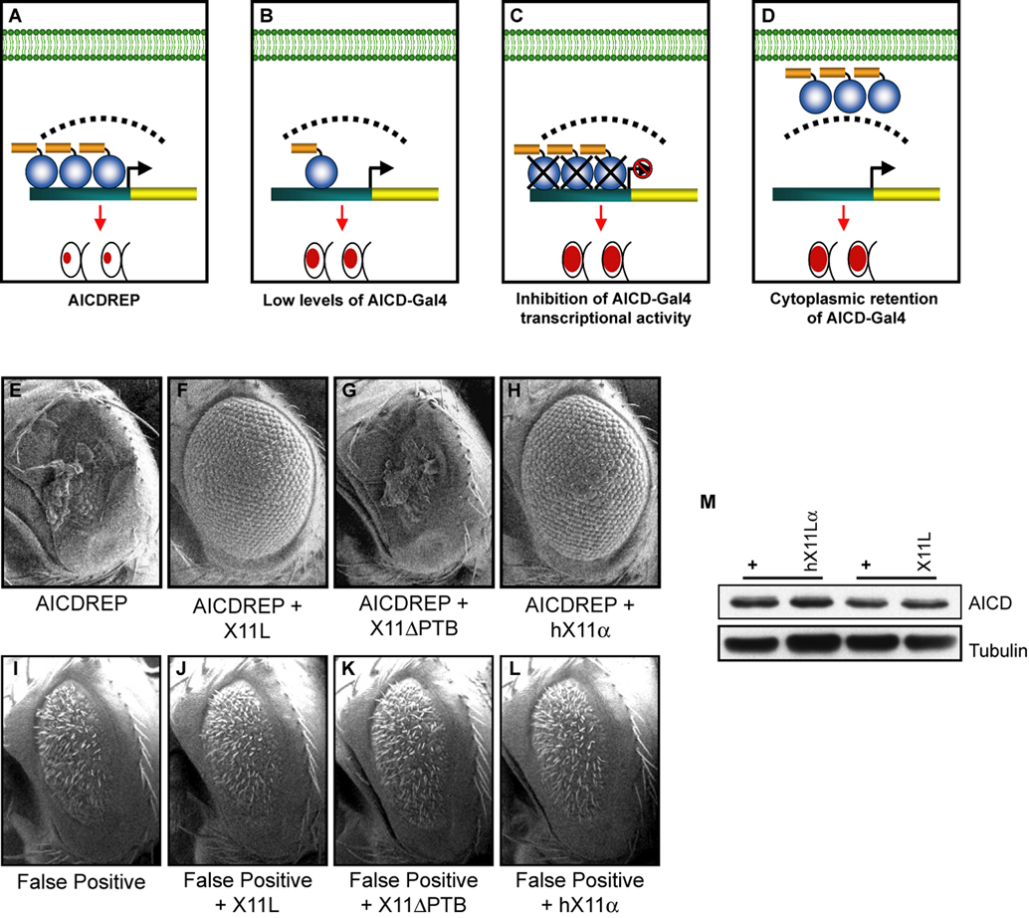
*X11L* overexpression suppresses the AICDREP. (A–D) Schematic illustration of the AICDREP. (A) AICDREP (GMR-AICD-Gal4, UAS-*hid*) is composed of two transgenic components. One expresses a fusion protein (AICD-Gal4) (yellow bar connected to blue circle) specifically in the eye. The other expresses the apoptotic gene *hid* (yellow bar) under the control of the UAS promoter (green bar). The AICDREP flies exhibit almost no eye, due to extensive apoptosis. The AICDREP serves as a useful system to identify genes that regulate AICD levels (B), transcriptional activity (C), as well as AICD cytoplasmic retention (D) in vivo. False positive modifiers such as factors regulating the expression levels of transgenes, Gal4's ability to activate transcription, or apoptosis can be excluded using GMR-Gal4, UAS-*hid* (I). (E–L) SEMs of adult fly eye of various genotypes. (E) The AICD reporter flies exhibit an almost total lack of ommatidia. (F) Overexpression of *X11L* results in a dramatic suppression of the AICDREP and restores the eye to nearly wildtype. (G) Overexpression of X11ΔPTB fails to suppress the AICDREP phenotype. (H) Expression of human X11α also demonstrates dramatic rescue of the AICDREP eye phenotype. (I–L) Overexpression of X11L, X11ΔPTB or human X11α does not affect the eye phenotype of GMR-Gal4, UAS-*hid* transgene. (M) Western Blotting of adult head lysates. Neither X11L overexpression nor X11α expression alters AICD levels. Each experimental and its relevant control samples are linked by a horizontal line.

To explore if *X11L* overexpression regulates AICD function independently of γ-secretase regulation, we generated transgenic flies that overexpressed a fusion protein of the AICD and Gal4 specifically in the eye (GMR-AICD-Gal4). Flies carrying both GMR-AICD-Gal4 and UAS-*hid* transgenes (Fig. 4A and 4E, hereafter called the AICDREP) were viable but exhibited an almost complete absence of eye tissue, due to extensive apoptosis of the cells in the eye [62]–[64]. This AICDREP (Fig. 4E) displayed a much smaller eye size than that of GAMAREP (Fig. 2C), presumably because in the AICDREP, Gal4 is not tethered to the membrane as compared to C99-Gal4, and therefore does not require any liberation by γ-secretase. The AICDREP thus serves as a useful system to identify genes that regulate AICD function in vivo (Fig. 4B–D). Remarkably, overexpression of *X11L* in the presence of AICDREP almost completely restored the eye size to wildtype (Fig. 4F). This suggests that the major effect of *X11L* overexpression is the γ-secretase independent inhibition of AICD. In addition, expression of human *X11α* suppressed the AICDREP as effectively as overexpression of *X11L* (Fig. 4H), indicating functional conservation between *Drosophila* and human *X11*.

Mammalian X11α has been shown to bind to the AICD via its PTB domain [30], [31]. If X11L overexpression exerts its major effect on AICD, we would expect that expression of a truncated X11L lacking its PTB domain (X11LΔPTB) should no longer be able to suppress the GAMAREP or AICDREP phenotypes. Indeed, eye-specific overexpression of X11LΔPTB (Fig. 2K) failed to suppress both GAMAREP and the AICDREP (Fig. 2L and 4G), further supporting the hypothesis that X11L regulates AICD function. Importantly, neither overexpression of X11L, X11LΔPTB nor human X11α affected the GMR-Gal4, UAS-*hid*)\ eye phenotype (Fig. 4I-L), suggesting that X11 expression acts specifically on AICD. Furthermore, the expression of X11L or X11α did not affect AICD steady-state levels (Fig. 4M), suggesting that X11 may promote cytoplasmic retention of AICD, or inhibit its ability to regulate transcription.

### *X11L* overexpression does not affect the γ-secretase mediated cleavage of Notch

**Figure 5:**
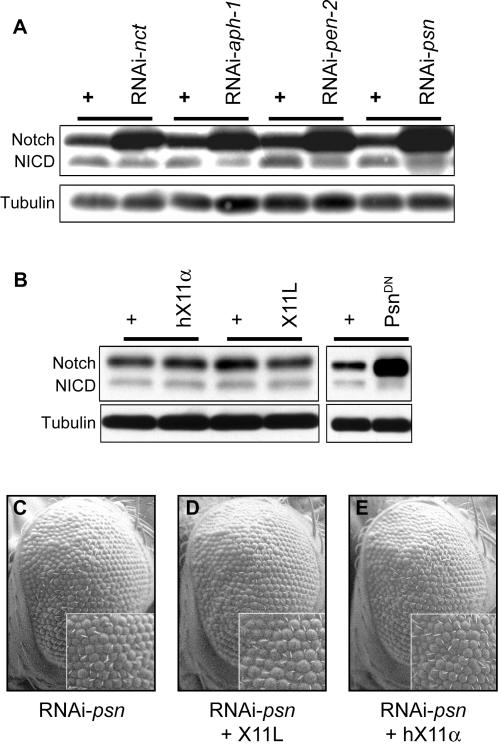
*X11L* overexpression does not regulate the effect of γ-secretase activity on Notch. (A–B) Western blotting of adult head lysates from various transgenic flies expressing the S2-cleaved Notch fragment (a γ-secretase substrate). (A) Eye-specific silencing of any of the four component of the γ-secretase complex results in a reduction of NICD levels and an increase in the levels of uncleaved Notch. (B) Overexpression of either *X11L* or human *X11α* fails to alter S2-cleaved Notch levels or NICD levels, in contrast to expression of Psn^DN^. (C–E) SEMs of adult fly eyes of various genotypes. Eye-specific expression of either *Drosophila X11L* (D) or human *X11α* (E) does not modify the eye phenotype due to eye-specific knockdown of *psn* (C). Insets show a close-up view of the ommatidia.

Our observation that overexpression of fly and human *X11* does not alter γ-secretase mediated cleavage of APP stands in contrast to a previous report [36]. To explore this issue further, we asked whether *X11L* overexpression could regulate γ-cleavage of Notch, another substrate of γ-secretase. Notch is a transmembrane receptor that mediates cell-cell communication events in multiple developmental contexts [65], [66]. Following cleavage of its extracellular domain (the S2 cleavage), Notch requires γ-secretase-mediated cleavage (denoted S3) to release the Notch intracellular domain (NICD), and activate downstream signaling events [67]. To analyze Notch processing, a transmembrane version of Notch representing the S2 cleaved fragment was Myc-tagged at its C-terminus and specifically expressed in the fly eye. Silencing of any one of the four γ-secretase components led to decreased γ-cleavage of Notch, as demonstrated by decreased levels of NICD, and an increase in the levels of uncleaved Notch (Fig. 5A). Overexpression of Psn^D279A^ also caused reduced levels of NICD and an accumulation of uncleaved Notch (Fig. 5B). In contrast, neither *X11L* overexpression nor expression of human *X11α* led to any significant effects on NICD production (Fig. 5B).

To further test the hypothesis that overexpression of *X11L* does not affect γ-secretase activity, we searched for a genetic interactions between *X11L* and *psn* in vivo. RNAi-mediated silencing of *psn* specifically in the eye (GMR-RNAi-*psn*) resulted in flies with small, rough eyes (Fig. 5C). This is likely due to impaired cleavage of Notch since partial loss of *Notch* function also results in small, rough eyes [68]. As expected, overexpression of *psn* partially suppressed the rough eye phenotype associated with GMR-RNAi-*psn*, while expression of Psn^D279A^ enhanced this phenotype (data not shown). In contrast, expression of either *Drosophila X11L* or human *X11* did not result in enhancement of the GMR-RNAi-psn eye phenotype (Fig. 5D and 5E), suggesting that overexpression of *X11L* does not significantly inhibit γ-secretase activity in vivo.

### *ubqn* acts to stabilize the steady-state levels of APP

**Figure 6:**
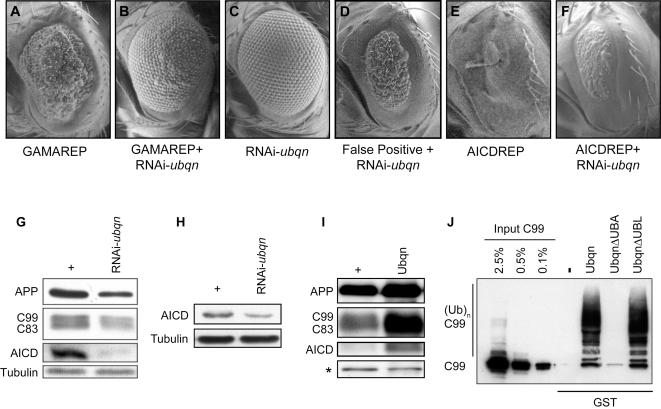
Ubqn binds to APP via its UBA domain and modulates APP levels. (A–F) SEMs of adult fly eyes of various genotypes. Eye-specific *ubqn* knockdown by itself does not show any visible phenotypes (C). However, silencing of *ubqn* strongly suppresses the GAMAREP phenotypes (compare B to A). *ubqn* knockdown does not modify the eye phenotype of the false positive reporter (GMR-Gal4, UAS-*grim*/*reaper*) (see Fig. 2D). (D). Eye-specific silencing of *ubqn* modestly suppresses the eye phenotype of AICDREP (compare F to E). (G–I) Western blotting of adult head lysates. (G) Eye-specific silencing of *ubqn* leads to decreased levels of APP, CTFs and AICD. (H) *ubqn* knockdown results in decreased AICD-Myc levels. (I) Eye-specific overexpression of *ubqn* results in an increase in the levels of APP, the CTFs and AICD. A non-specific band (marked by an asterisk) serves as the loading control. (J) GST pulldown assay. The input lanes show C99 levels from 0.1 to 2.5% of the S2 cell lysates used for each pulldown. Compared with control (GST alone), Ubqn and UbqnΔUBL, but not UbqnΔUBA, binds to C99. In addition, Ubqn and UbqnΔUBL show similar binding to C99, as well as to higher molecular weight products that are likely polyubiquitinated forms of C99.

To study *ubqn* function in *Drosophila*, we generated two RNAi constructs targeted to two independent regions of *ubqn* (the coding region and the 3′-untranslated region, respectively), and used these transgenic flies to carry out tissue-specific silencing [47]. As described previously, ubiquitous expression of RNAi-*ubqn* resulted in a loss of detectable Ubqn protein [47]. Both *ubqn* RNAi transgenes silenced *ubqn* expression to similar levels and gave identical phenotypes in all experiments [47]. Using the same RNAi hairpins, we carried out eye-specific silencing of *ubqn* (GMR-RNAi-*ubqn*); effects confirmed by Western blotting of head lysates using anti-Ubqn antibodies (Supplementary Fig. S1). Though silencing of *ubqn* function did not lead to any overt developmental phenotypes (Fig. 6C), it strongly suppressed the GAMAREP phenotype (Fig. 6B compared with 6A). Importantly, silencing of *ubqn* did not alter the false positive reporter eye phenotypes (Fig. 6D compared with 2D), suggesting that the suppression of GAMAREP was specifically due to an effect on C99.

We next investigated how *ubqn* loss-of-function suppressed GAMAREP. APP-Myc was co-expressed with human BACE in the eye. Silencing of *ubqn* resulted in a reduction of steady state levels of full length APP as well as its proteolytic fragments (C99, C83 and AICD) in the eye (Fig. 6G). Importantly, the reduction of full length APP and its cleaved forms occurred largely proportionately (Fig. 6G), suggesting that loss of *ubqn* leads to a decrease in the overall steady-state levels of APP, not a decrease in the activity of any particular secretase.

Since silencing of *ubqn* resulted in a decrease in AICD levels as a consequence of reduced APP levels, we asked if silencing *ubqn* would also affect the AICD reporter eye phenotypes. RNAi-*ubqn* flies were crossed to flies overexpressing Myc-tagged AICD. Indeed, we observed that eye-specific expression of RNAi-*ubqn* led to a modest suppression of the AICDREP (Fig. 6F compared with 6E), and a reduction in AICD levels in vivo (Fig. 6H).

To determine if *ubqn* acts as a dose-dependent regulator of APP levels, we examined the consequences of *ubqn* overexpression on APP levels. Eye-specific overexpression of *ubqn* (GMR-*ubqn*) did not generate any overt eye phenotypes, or suppress the GAMAREP eye phenotypes (data not shown). Expression levels of *ubqn* overexpression was confirmed by anti-Ubqn antibodies (Supplementary Fig. S1). However, *ubqn* overexpression (GMR-Gal4, UAS-*ubqn*) led to an increase in APP levels (Fig. 6I). As with silencing of *ubqn*, *ubqn* overexpression increased the steady state levels of the full length and γ-secretase cleaved fragments of APP largely in proportion, suggesting that perturbation of Ubqn levels does not affect γ-cleavage of APP (Fig. 6I). Together, we concluded that the suppression of the GAMAREP phenotype caused by loss of *ubqn* function is most likely the result of a decrease in APP steady-state levels. While loss of *ubqn* strongly suppressed the GAMAREP phenotype, we did not observe a significant enhancement of GAMAREP upon *ubqn* overexpression. This is most likely due to the fact that our GAMAREP reporter is much less sensitive in detecting enhancers than suppressors. This is based on multiple screens carried out to identify modifiers of GAMAREP, from which we isolated a dozen GAMAREP suppressors but no enhancers (unpublished observations).

How Ubqn regulates APP levels is unclear, but could involve direct interactions between these proteins. To explore this hypothesis, we asked if Ubqn can bind to APP directly. We mixed purified GST and GST-Ubqn fusion proteins with lysates from Schneider 2 (S2) cells transfected with Myc-tagged C99. Indeed, Myc-C99 specifically bound GST-Ubqn, but not GST alone (Fig. 6J). Interestingly, in addition to native-sized C99, GST-Ubqn preferentially bound to high molecular weight products, which are likely polyubiquitinated forms of C99 (Fig. 6J). Deletion of the UBA domain, but not the UBL domain, significantly abrogated the interaction between Ubqn and C99 (Fig. 6J), indicating that the UBA domain is required for this interaction.

## Discussion

Here, we report the identification of two APP regulators, X11L and Ubqn, using a living reporter, GAMAREP. GAMAREP allows us to effectively identify regulators of γ-secretase activity, APP steady-state levels and/or AICD function. Both the steady-state levels and the processing of APP have been demonstrated to be important in the pathogenesis of AD. However, the function of AICD, a much less studied APP fragment, may also be crucial for AD pathogenesis, since AICD is generated along with Aβ and may have functions including transcriptional regulation, signaling, apoptosis and calcium homeostasis. Importantly, when a protein regulates multiple aspects of APP, either directly or indirectly, the combination of GAMAREP, AICDREP, and our in vivo cleavage assays allows us to uncover the relative contributions of these regulatory inputs. This is of particular relevance in this study since both X11 and UBQLN1 have been proposed to have multiple functions affecting myriad pathways in various systems. Therefore, it is important to determine in vivo which pathways play predominant roles in events related to AD pathogenesis. Our data suggest that the predominant role of X11L is to regulate APP at the level of the AICD by a process requiring its PTB domain, whereas the major role of Ubqn is to regulate APP levels, likely through a direct physical interaction with APP that is dependent on the UBA domain of Ubqn.

As a modifier of AICDREP, X11L does not regulate AICD levels. Following γ-cleavage, AICD is thought to migrate into the nucleus. It is likely that *X11L* overexpression causes cytoplasmic retention of AICD based on the following observations. In mammalian cultured cells, *X11* overexpression can reduce nuclear localization of the AICD with Tip60 [69]. Moreover, X11 can shuttle between the nucleus and cytoplasm to regulate AICD transcriptional targets [70]. Our functional studies using AICDREP suggest that the PTB domain of X11L is essential for X11's ability to regulate AICD. Therefore, our hypothesis is that *X11L* overexpression leads to the cytoplasmic retention of AICD via a direct physical interaction between the PTB domain of X11L and AICD.

We have also showed that, in contrast to prior studies [36], *X11L* overexpression does not inhibit γ-secretase activity in vivo. In *Drosophila*, none of the APP cleavage products, C83, C99 or AICD, is affected by *X11L* overexpression. Moreover, *X11L* overexpression does not inhibit γ-secretase activity in vivo towards another key substrate, Notch, at the level of cleavage, or at the level of genetic interaction with *psn*. In a prior study, King et al concluded that X11 inhibits γ-secretase activity, based largely on the findings that extracellular Aβ levels were decreased upon *X11* overexpression [36]. They interpreted this to be due to decreased Aβ production by γ-secretase inhibition. However, decreased extracellular Aβ levels could also result from decreased Aβ secretion or increased Aβ degradation. In fact, another in vivo study suggested that although *X11* overexpression led to reduced Aβ levels, levels of C83 and C99 (direct γ-secretase substrates) did not increase [29]. Since overexpression of *X11* can increase the steady-state levels of APP, likely by modulating APP trafficking to certain compartments in the secretory pathway [25], [27], [33], [34], an alternative interpretation of work by King et al is that *X11* overexpression decreases Aβ via its modulation of APP trafficking, rather than by regulating γ-secretase cleavage. Therefore, these prior studies are, in fact, consistent with our data and interpretation.

Our data also suggest that the predominant function of *ubqn* is to regulate APP levels, not γ-secretase activity. We have established that Ubqn can bind to and stabilize APP. In contrast to the selective reduction of AICD and increase in CTFs seen following the knockdown of any one of the four γ-secretase components, both *ubqn* loss-of-function and *ubqn* overexpression modify the levels of APP and its cleavage fragments largely in proportion. Our results are consistent with findings made by Zhang et al and different from Hiltunen et al; possible reasons for these differences are discussed in Zhang et al [50].

In this study, we did not observe any alterations in γ-secretase activity on APP in response to the silencing of *ubqn* or *ubqn* overexpression, similar to observations made by others [48]–[50]. However, we have previously shown that Ubqn binds to Psn and antagonizes *psn* function both during development and during adult-onset eye neurodegeneration in *Drosophila* [47]. There are several ways these observations can be reconciled. γ-secretase activity is known to vary depending upon the substrate and the tissue in which it acts [71]. It is possible that *ubqn* antagonizes *psn* activity such that its effect on γ-secretase is more evident on substrates other than APP in the eye. As a result, the modification of the γ-cleavage of APP by *ubqn*, if any, might be below the detection limits of our assay. Alternatively, it is well established that in addition to being the catalytic core of the γ-secretase complex, Psn can also function in a γ-secretase independent fashion [72], [73]. Therefore, it is possible that *ubqn* primarily inhibits γ-secretase independent functions of *psn*, leaving γ-secretase dependent activity largely intact. In either case, the primary effect of *ubqn* on APP appears to be the regulation of APP steady state levels, and not inhibition of the γ-cleavage of APP.

Here, using GAMAREP and AICDREP, in conjunction with in vivo cleavage analysis, we have identified factors that regulate APP. Further screens using these tools are likely to identify other proteins which may have implications for AD pathogenesis. Since increased expression of X11 attenuates Aβ production or secretion in APP transgenic mice [28], [29], but does not act on γ-secretase directly (this work), X11 may present an attractive therapeutic target for AD. Similarly, since reduced Ubqn levels result in a modest decrease in APP levels, whereas expression of both human wildtype and the AD variant of UBQLN1 leads to adult onset, age-dependent eye degeneration in *Drosophila* [47], UBQLN1 may also be considered as a possible drug target for AD.

## Materials and Methods

### Molecular Biology

A microRNA-based technology [74] was used for RNAi silencing. To silence *psn, nct, aph-1* and *pen-2*, the respective coding regions were independently targeted. PCR products of these microRNA precursors were cloned into pGMR. To generate GMR-*X11L* and UAS-*X11L*, the *X11L* coding sequence was PCR amplified from the EST clone, LD29081, and subcloned into each vector. To generate GMR-human-*X11α,* a clone of *X11α* (kindly provided by Declan McLoughlin) was subcloned into the EcoR1 and Not1 sites of pGMR. To generate GMR-*X11*ΔPTB, the insert of UAS-X11LΔPTB [75] was subcloned into pGMR vector. To generate GMR-AICD-Gal4, the nucleotide region encoding the predicted human AICD fragment was PCR amplified and fused in-frame, upstream of an *S. cerevisiae* Gal4 sequence. To make GMR-*ubqn*, GMR-RNAi-*ubqn*CDS and GMR-RNAi-*ubqn*UTR, the inserts from UAS-*ubqn*, UAS-RNAi-*ubqn*CDS and UAS-RNAi-*ubqn*UTR [47] were subcloned into pGMR vector, respectively. To generate pGMR-APP-9Myc and GMR-AICD-9Myc, coding sequence of APP_695_ or AICD was PCR amplified and cloned in-frame upstream of a 9xMyc sequences that has been previously subcloned into pGMR vector. C99-9Myc was also generated by PCR and subcloned to generate pMT-C99-9Myc and GMR-C99-9Myc, respectively. To make pGEX-UbqnΔUBL, a PCR fragment encoding UbqnΔUBL was subcloned into the EcoR1 and Not1 sites of the modified pGEX4T-1 vector. pGEX-*Ubqn* and pGEX-*Ubqn*ΔUBA, were described previously [47]. All cloned PCR products were confirmed by DNA sequencing.

### *Drosophila* Genetics and Strains

For the X chromosome EP screen, individual lines of females carrying a single inserted EP element were individually crossed to GAMAREP males. Progeny carrying one copy of an EP insertion and one copy of GAMAREP were examined, and their eye size compared with control flies derived from a cross of *w^1118^* females to GAMAREP males. For experiments involving transgenic flies, multiple independent fly lines were generated (Rainbow Transgenic Flies) and tested for each transgene. UAS-*BACE* flies [60] were obtained from Rita Reifegerste via Doris Kretzschmar, and UAS-*hid* flies [64] were obtained from Bruce Hay. GMR-Psn^D257A^ was described previously [59].

### Scanning Electron Microscopy

Freshly sacrificed flies were mounted on their side with one eye upward on white tape using clear nail polish. All flies were placed on a rotating platform to permit for orientation under vacuum and were imaged at 180× magnification and 100 psi using a Hitachi 2460N scanning electron microscope. Analysis of eye phenotypes was performed as described previously [59].

### Antibody Generation

A fusion of GST-TEV to Drosophila X11L residues 452–775, corresponding to a region within the N-terminal domain, with an intervening TEV protease recognition site, was purified from *E. coli* lysates. Glutathione agarose-retained proteins were cleaved by TEV protease to remove GST, and used to immunize rabbits (Imgenex).

### Lysate Preparation and Western Blotting

Heads from age and sex-matched adults were disrupted in lysis buffer, complete protease inhibitor cocktail (Roche) using a sonicator-3000 from MISONIX. Samples were sonicated. Samples were boiled, centrifuged, and total protein from 4 heads per genotype was analyzed by Western blotting. Antibodies used were anti-Myc (Upstate), anti-Ubqn [47], anti-X11L and anti-Tubulin (Sigma).

### S2 Cell Culture and Transfection

S2 cells were grown in Schneider's *Drosophila* medium (Invitrogen) supplemented with 10% FBS, 50 units/ml penicillin, and 50 μg/ml streptomycin at room temperature. Transfections were carried out using MaxFect transfection reagent (Molecula). Typically, 1.5×10^6^ cells plated in a 12-well dish were transfected with 0.7 – 1 μg total plasmid DNA plus 5 μL MaxFect reagent. Metallothionein promoter expression was induced with 0.5 mM copper sulfate 24 hours after transfection. S2 cell lysates were prepared by harvesting transfected cells (∼5×10^6^) in 1 mL lysis buffer, followed by incubation on ice for 10 minutes and centrifugation (16,000×g, 10 min) to pellet insoluble debris.

### GST Pulldown Assay

10 μg GST fusion proteins purified from E. coli lysates were retained on 15 μL glutathione beads, and mixed with 400 μg S2 cell lysate in 1mL total volume. Retained proteins were eluted by boiling in Laemmli sample buffer, and detected by Western blotting with anti-Myc antibody (Covance) [47].

## Supporting Information

Figure S1 The expression levels of X11L and ubqn transgenes. (A) We have generated polyclonal antibodies against X11L. X11L overexpression is accomplished using either an eye-specific promoter (GMR-X11L) or the eye-specific driver using the UAS-GAL4 system (GMR-Gal4, UAS-X11L). Western blots of lysates from fly heads overexpressing X11L or lysates from Schneider 2 cells overexpressing X11L (MT-X11L) reveal a band of predicted size using anti-X11L antibodies. (B) Western blots of lysates from fly heads expressing RNAi-ubqn or ubqn using anti-Ubqn antibodies. Silencing of ubqn significantly reduces Ubqn levels, while ubqn overexpression increases Ubqn levels. A non-specific band (*) serves as protein loading control.
(1.06 MB TIF)
