# Supplementary figures and images for: Role of X11 and ubiquilin as In Vivo Regulators of the Amyloid Precursor Protein in Drosophila
Source: PLoS One. 2008 Jun 25;3(6):e2495. doi: 10.1371/journal.pone.0002495 (PMC2429963; doi:10.1371/journal.pone.0002495)

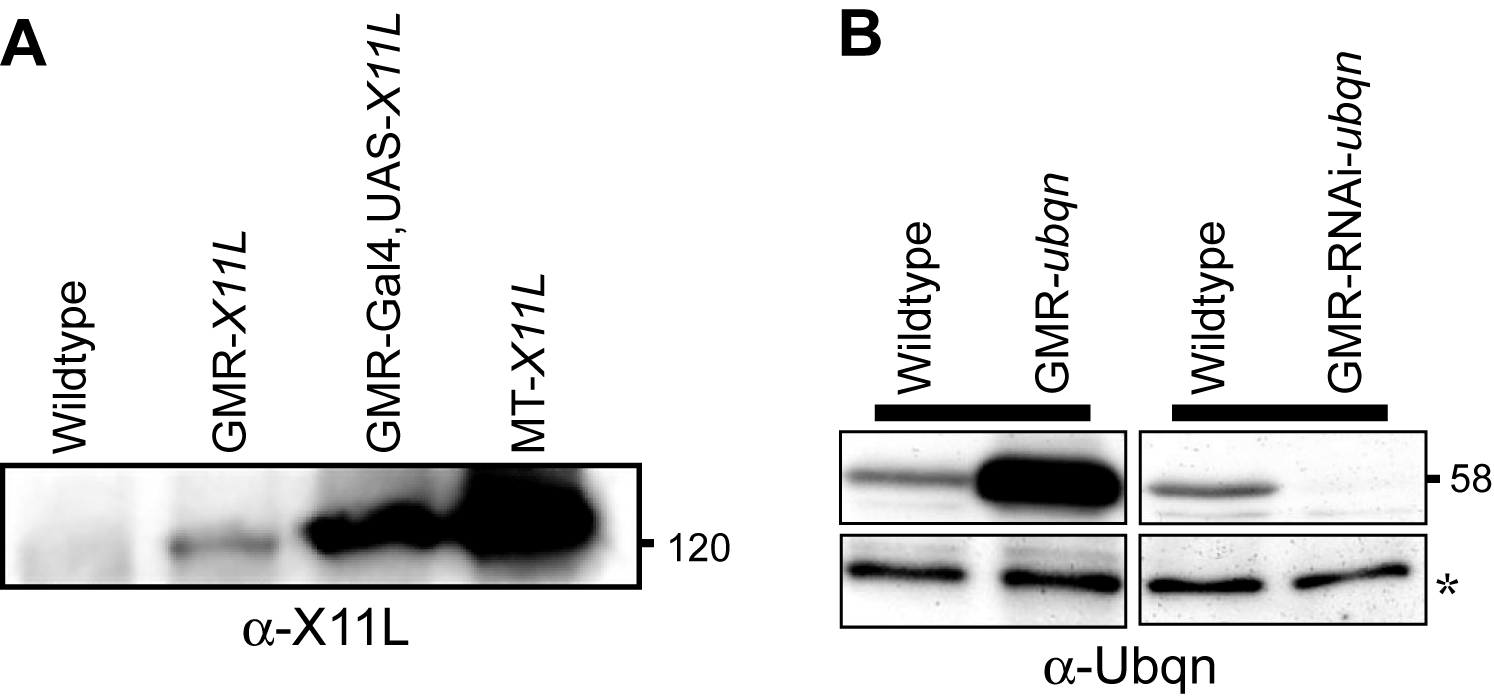

Supplement: Figure S1 — The expression levels of X11L and ubqn transgenes. (A) We have generated polyclonal antibodies against X11L. X11L overexpression is accomplished using either an eye-specific promoter (GMR-X11L) or the eye-specific driver using the UAS-GAL4 system (GMR-Gal4, UAS-X11L). Western blots of lysates from fly heads overexpressing X11L or lysates from Schneider 2 cells overexpressing X11L (MT-X11L) reveal a band of predicted size using anti-X11L antibodies. (B) Western blots of lysates from fly heads expressing RNAi-ubqn or ubqn using anti-Ubqn antibodies. Silencing of ubqn significantly reduces Ubqn levels, while ubqn overexpression increases Ubqn levels. A non-specific band (*) serves as protein loading control. (1.06 MB TIF) [file pone.0002495.s001.tif]
